# The Genomic Landscape in Philadelphia-Negative Myeloproliferative Neoplasm Patients with Second Cancers

**DOI:** 10.3390/cancers14143435

**Published:** 2022-07-14

**Authors:** Chia-Chen Hsu, Ying-Hsuan Wang, Yi-Yang Chen, Ying-Ju Chen, Chang-Hsien Lu, Yu-Ying Wu, Yao-Ren Yang, Hsing-Yi Tsou, Chian-Pei Li, Cih-En Huang, Chih-Cheng Chen

**Affiliations:** 1 Division of Hematology and Oncology, Department of Medicine, Chang Gung Memorial Hospital, Chiayi 61363, Taiwan; loofah1008@cgmh.org.tw (C.-C.H.); shang0320@cgmh.org.tw (Y.-H.W.); 8902025@cgmh.org.tw (Y.-Y.C.); agrb7289@cgu.edu.tw (Y.-J.C.); q12014@cgmh.org.tw (C.-H.L.); yuyun5801@cgmh.org.tw (Y.-Y.W.); b9802046@cgmh.org.tw (Y.-R.Y.); a6986a@gmail.com (H.-Y.T.); air110202@cgmh.org.tw (C.-P.L.); 2 College of Medicine, Chang Gung University, Tao-Yuan 33302, Taiwan

**Keywords:** myeloproliferative neoplasms, second cancers, whole exome sequencing, genetic predisposition, inflammation, *KRT6A*, driver mutation

## Abstract

**Simple Summary:**

Philadelphia-chromosome-negative myeloproliferative neoplasms (MPNs) are a group of clonal hematopoietic stem cell disorders characterized by the excessive production of differentiated myeloid cells. Other than the well-known propensity for leukemia transformation, MPN patients are also prone to developing second cancers (SCs) that arise from different embryonic dermal origins. This brings up an intriguing question: what is the molecular background leading to the development of SCs in MPN patients? To explore further, we used whole exome sequencing to characterize the genomic landscapes in 26 paired MPN samples stratified by the presence or absence of SCs. We found that mutated genes in MPN–SC samples were enriched in some critical immune-related pathways and inflammatory networks, an observation further supported by the increased plasma levels of cytokines. Our work provides the genomic landscape that profiles the genetic basis for SC tumorigenesis in MPN patients, and also demonstrates that inflammation could be indispensable in MPN–SC pathogenesis.

**Abstract:**

Patients with myeloproliferative neoplasms (MPNs) are characterized by systemic inflammation. With the indolent nature of the diseases, second cancers (SCs) have emerged as a challenging issue in afflicted patients. Epidemiological studies have confirmed the excessive risk of SCs in MPNs, but little is known about their molecular basis. To explore further, we used whole exome sequencing to explore the genetic changes in the granulocytes of 26 paired MPN patients with or without SC. We noticed that MPN–SC patients harbor genomic variants of distinct genes, among which a unique pattern of co-occurrence or mutual exclusiveness could be identified. We also found that mutated genes in MPN–SC samples were enriched in immune-related pathways and inflammatory networks, an observation further supported by their increased plasma levels of TGF-β and IL-23. Noteworthily, variants of *KRT6A*, a gene capable of mediating tumor-associate macrophage activity, were more commonly detected in MPN–SC patients. Analysis through OncodriveCLUST disclosed that *KRT6A* replaces *JAK2*V617F as the more prominent disease driver in MPN–SC, whereas a major mutation in this gene (*KRT6A* c.745T>C) in our patients is linked to human carcinoma and predicted to be pathogenic in COSMIC database. Overall, we demonstrate that inflammation could be indispensable in MPN–SC pathogenesis.

## 1. Introduction

Classical Philadelphia-negative (Ph-neg) myeloproliferative neoplasms (MPNs) are a group of clonal hematopoietic disorders—namely polycythemia vera (PV), essential thrombocythemia (ET), primary myelofibrosis (PMF), and pre-fibrotic myelofibrosis (pre-PMF)—that are characterized by the excessive production of differentiated myeloid cells [1]. Mutually exclusive driver mutations in *JAK2*, *CALR*, and *MPL* genes result in constitutive activation of Janus kinase-signal transducer and activator of transcription (JAK/STAT) signaling, which represents a hallmark of MPNs and leads to the development and progression of the diseases [2]. Clinically, these diseases are frequently complicated by troublesome constitutional symptoms, thromboembolic events, and a propensity for transformation into acute myeloid leukemia (AML). However, AML is by no means the only catastrophic malignancy co-occurring in MPNs, as multiple retrospective reports also demonstrate that MPN patients are prone to developing lymphoproliferative disorders and second cancers (SCs) [3,4,5,6,7]. While a high hazard ratio (HR) of 46.0 for developing AML was observed in MPN patients in a large, population-based cohort study, there were significantly increased risks for lymphoma and non-hematological SCs in these patients as well, as the reported HRs were 2.6 and 1.6, respectively [6].

Secondary AML arising from MPNs is a well-recognized consequence that commonly ensues cumulative, collaborating genetic and epigenetic events. The molecular features of secondary AML transformation have been studied thoroughly. Compared to de novo disease, post-MPN AML is more likely to be provoked by aberrant chromosomal changes, including complex karyotypes, monosomies, and 17p deletion, and mutations in epigenetic regulators, such as *IDH1/2*, *TET2*, *ASXL1*, and *EZH2* [8]. These genetic abnormalities, along with some other adverse clinical as well as laboratory features, constitute the main risk factors that prognosticate the development of secondary AML [8,9,10,11]. Given the overall dismal outcome of post-MPN AML, integrating clinical profiles with molecular signatures for personalized therapeutic strategies in MPN patients to avert leukemic transformation has been strongly advocated [12].

In contrast to what we know about the genetics and consequences of secondary AML in MPNs, the issue on the association between non-hematological SCs and MPNs is less thoroughly explored. Although population-based epidemiologic studies have confirmed the excessive risk of SCs in patients with MPNs [6,7,13,14], little is known about the molecular basis of their causal relationship. Systemic inflammation, a hallmark of MPN, has been widely regarded as the culprit of MPN-associated SC [15]. Whether additional mutations or molecular aberrancies in MPN cells reinforce the inception and propagation of SC remains to be elucidated.

To delineate the genetic background of an SC in MPN, we performed whole exome sequencing (WES) in the granulocytes of a cohort of MPN patients with SCs. Comparative results were obtained in an age-, gender-, and subtype-matched and driver-mutation-matched group of MPN control patients who did not have SCs. The results provided us a comprehensive overview characterizing the molecularly distinct genetic changes in MPN patients with SCs.

## 2. Materials and Methods

### 2.1. Patient Source, WES, and Data Analysis

The study was approved by the Institutional Review Board (IRB) of our hospital (IRB approval number: 202001099B0). Processing of peripheral blood (PB) samples from these patients, isolation of granulocytes, and detection of the driver mutations in them were performed as described previously [16,17].

**Figure 1:**
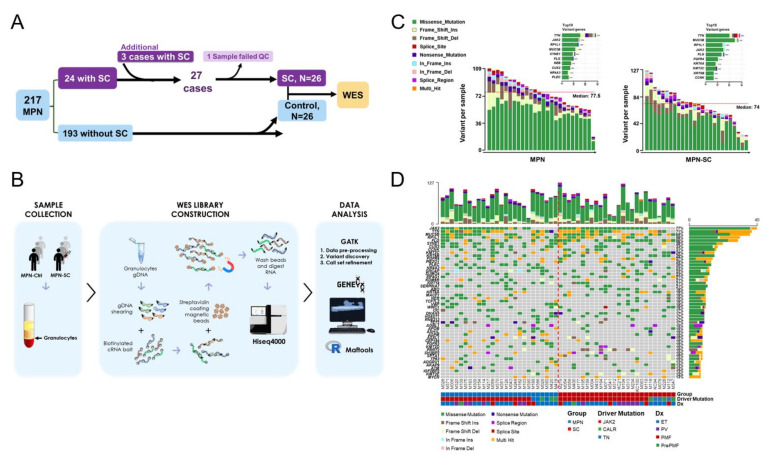
Patterns of genomic variations in paired MPN patients with or without SCs. (**A**) Flow chart on the enrollment of 26 MPN control cases and 26 MPN patients with SCs. (**B**) Diagrammatic illustration of the experimental procedures of WES in this study. (**C**) Comparing data on genomic variations between MPN control cases (left) and MPN patients with SCs (right). Subtypes of genomic variants (such as indel, frameshift, and missense or nonsense mutations) were depicted and represented by different color boxes. (**D**) Genes with variations in individual patients were highlighted based on different sub-categorizing strategies. Bottom column, first row: stratification based on the absence (control group, blue boxes, mainly on the left) or presence (study group, red boxes, mainly on the right) of an SC. Bottom column, second row: stratification based on driver mutations: *JAK2* in red, *CALR* in green, and triple negative (TN) in blue. Bottom column, third row: stratification based on MPN subtypes: ET in blue, PV in purple, PMF in red, and prePMF in green.

The enrollment of candidate patients and the experimental procedures of WES are depicted in Figure 1A,B. To begin with, following DNA extraction with Trizol-Alcohol precipitation, Qubit Fluorometric Quantification (Thermo Fisher Scientific Inc., Waltham, MA, USA) and Bioanalyzer (Agilent, Santa Clara, CA, USA) were used to assess the quality and quantity of DNA content. The WES libraries were constructed using Agilent’s SureSelect Human All Exon V6 Kit (Agilent), which covered nearly 60 Mb of the exon region. Briefly, one microgram of genomic DNA (gDNA) was used for adapter-ligated amplification, and sample purification was performed using the Agencourt AMPure XP beads (Beckman Coulter, Brea, CA, USA). As per the manufacturer’s protocol, 750 ng of each gDNA library was hybridized with capture baits for 24 h at 65 °C and Dynabeads MyOne Streptavidin T1 (Thermo Fisher Scientific Inc.) was used to capture the hybridized DNA library. Through post-hybridization amplification, sample index tags were added and the final DNA products were purified with AMPure XP beads. All gDNA libraries were analyzed on TapeStation 4200 with D1000 screen-tape for quality control. The constructed libraries were then subjected to analysis on the Illumina HiSeq X platform (Illumina, San Diego, CA, USA) using HiSeq X Reagent Kits with 150 bp paired-end reading length.

FastQC and multiqc [18] were used to appraise the quality of nucleotide reads upon obtaining raw data. We also applied trimmomatic [19] to remove the adaptor sequences and low-quality reads. The generated clean reads were aligned through the Burrows–Wheeler Alignment (BWA) tool [20] using the UCSC hg19 genome version, and the output binary alignment and map (BAM) file was then subjected to analysis with qualimap [21] and picard, tools for evaluating reading depth, coverage, and duplication percentage. Following reads mapping, HaplotypeCaller from the Genome Analysis Toolkit (GATK) [22] and SnpEff [23] were used to reduce miscalls from short insertions/deletions (InDels) as well as to minimize artifacts generated during sequencing by adjusting the quality score for each read. All acquired single-nucleotide variations (SNVs) and InDels were saved in a Variant Call Format (VCF) file and uploaded onto Geneyx Analysis™ (Geneyx Genomex, Ltd., Herzliya, Israel). Somatic SNVs were confirmed with MuTect2 in the GATK pipeline. Details of all SNVs and InDels were classified and annotated through ANNOVAR [24], InterVar [25], and SnpEff. Control-FREEC, in conjugation with ANNOVAR, was applied to identify copy number variations (CNVs).

Several criteria were adopted to appraise SNVs or InDels of somatic or germline mutations. The depth of reads must exceed 15, and only variants with an allele frequency of more than 15% were considered significant and included. All synonymous, intronic, and upstream and downstream variants were removed and excluded from further analysis. However, variants located at the splicing sites (i.e., between ±2 bps of a spicing site) were regarded as functional and retained. We also kept variants predicted to be deleterious in the Sorting Intolerance From Tolerant (SIFT) [26] analysis and those deemed to be probably or possibly pathogenic in the Polymorphism Phenotyping version 2 (PolyPhen-2) [27] tool. Following the filtering process, the preserved variant candidates were converted to Mutation Annotation Format (MAF) for further analysis. Maftools [28] was used for oncoplot visualization, somatic interaction, position-based cancer drivers analytics, and comparison between two cohorts. Variants were also annotated and assessed through ClinVar and Catalogue of Somatic Mutations in Cancers (COSMIC). For gene ontology, the Reactome was applied for functional analysis. To appraise genes with potential bias toward mutational clustering within the protein sequences, we also used the OncodriveCLUST algorithm [29] to assess coding-silent mutations.

### 2.2. Mutation Validation with Sanger Sequencing

To attest the findings obtained from WES, we performed Sanger sequencing on several target genes that exhibited differential mutational patterns between the two cohorts of patients. The genes and their primer sequences are listed in Table S5.

### 2.3. Measurement of Plasma Levels of Inflammatory Cytokines

To quantify plasma cytokine levels, the multiplex ELISA-based Q-Plex™ Human Cytokine HS Screen Array (Quansys Biosciences, Logan, Utah, USA) was used. The kit contains an array of inflammatory cytokines, including IL-1α, IL-1β, IL-2, IL-4, IL-5, IL-6, IL-10, IL-12p70, IL-13, IL-15, IL-17, IL-23, IFN-γ, TNF-α, and TNF-β. The assay was performed in a 96-well plate following the manufacturer’s protocol. The Q-View™ Imager Pro and Software were used for data capture and calculation of the final concentrations, respectively. Additionally, human TGF-β1 ELISA Kit (BOSTER Biological Technology, Pleasanton, CA, USA) was used to quantify plasma TGF-β levels. Briefly, the amount of TGF-β was determined at an optical density of 450 nm wavelength through the 800 TS Absorbance Reader (Bio-Tek instruments Inc., Winooski, VT, USA). The TGF-β concentrations of MPN samples were calculated through interpolation against a standard curve. In all samples, the plasma levels of various cytokines were analyzed in duplicates. Samples with low precision and repeatability (defined as a coefficient of variation of more than 10%) and those with apparent outlier values were excluded from subsequent statistics.

### 2.4. Genotyping for JAK2 46/1 Haplotype

SNP rs12340895 was used as a tag SNP to determine the *JAK2* 46/1 haplotype. PCR reactions with primers encompassing rs12340895 were performed as previously reported [30]. PCR products were sent for Sanger sequencing. The nucleotide G is associated with the 46/1 haplotype, while the nucleotide C is associated with the non-46/1 haplotype. Information on the *JAK2* 46/1 haplotype (rs12340895) in healthy Taiwanese population was obtained from the Taiwan Biobank database (which is publicly available at https://taiwanview.twbiobank.org.tw/; accessed on 7 March 2022). The SNP in the database were screened by WES using next-generation sequencing platforms of Proton (520 healthy subjects) and Illumina (1000 healthy subjects).

## 3. Results

### 3.1. MPN Patients with SCs Are Older but Do Not Exhibit Unique Clinical Characteristics

In our cohort of 217 MPN patients, we identified 24 cases (11.1%) with SCs. Three additional MPN patients with SCs, referred from other institutes for consultation on the diagnosis and management of MPNs, were also included. This made overall 27 cases (Figure 1A). The clinical profiles of these patients are summarized in Table S1. For the time sequence, MPNs and SCs were regarded as concurrent diseases if the diagnoses were made within 6 months of each other. In patients who had a “second cancer” antedating the diagnosis of MPN, we still considered these two events inter-correlated with each other as the clinical course of MPN might go unnoticed for quite some years before an accurate diagnosis can be made. Moreover, MPN could go through a lengthy prodromal phase or even manifest as subclinical clonal hematopoiesis of indeterminate potential, during which period some susceptible catastrophic events might occur [31].

We next compared the baseline characteristics between MPN patients with SCs (*n* = 27) and our whole MPN cohort who did not develop SCs (*n* = 193) (Table 1). It should not be surprising to see that those with SCs were significantly older than their counterparts (70.2 ± 14.6 vs. 60.8 ± 16.8, in years, *p* = 0.006). There was also a trend for a lower hemoglobin level in MPN patients with SCs, although the comparison did not reach statistical significance. Other clinical parameters were not drastically discrepant.

Based on the similarity between tumorigenesis and embryonic development of humans, we also subdivided second cancers into three different categories according to their derivative origins (ectoderm, mesoderm, and endoderm) (Table 1 and Table S1). A comparison between dermal origins and either MPN subtypes or driver mutations is listed in Table S2. Briefly, there was no apparent predilection for an MPN subtype co-occurring with tumors of a specific dermal origin. However, it is noteworthy that *JAK2*-mutated MPN patients were more likely to develop endodermal tumors, whereas tumors found in those with *CALR* mutation were more likely to be mesodermal origin (*p* = 0.027).

Taken together, our data indicate that an older age is the only factor segregating MPN patients with SCs from their counterparts with regard to the clinical characteristics. Different driver mutations are associated with distinct developmental dermal origins, but the limited case numbers in our study warrant further validation from other larger cohorts.

### 3.2. Patterns of Genomic Variations in MPN Patients with SCs Are Not strikingly Disparate from Those of Control Cases

We aimed to use WES to explore potential genetic predisposition to SCs in patients with MPNs. Among 27 granulocyte samples of MPN patients with SCs, one DNA sample failed quality control assessment and was removed. For the purpose of comparison, this work also involved 26 age-, gender-, and diagnosis-matched and driver-mutation-matched MPN patients without SCs as controls (Figure 1A; Table S3). Importantly, there were no differences between them with regard to prior cytotoxic agent exposure, prior duration of hydroxyurea therapy, and the length of follow-up time. Figure 1B outlines the whole experimental procedures of WES as well as how the data were analyzed.

To elucidate potential disparity between MPN patients with and without SCs, we first compared the genomic alteration profiles between MPN controls and MPN–SC cases. Figure 1C depicts the number and patterns of genomic variation in each individual patient. It is clear that the most common genomic change was missense mutation. The median number of variants was actually quite similar between the two groups (Figure 1C). Through highlighting changes in individual gene in each case (Figure 1D), we also found that there were no meaningful differences in the patterns of genetic variants either between the two groups of patients or among patients subcategorized by distinct driver mutations. Patients stratified by MPN subtypes did not exhibit unique features of genomic variations (Figure 1D) either.

**Figure 2:**
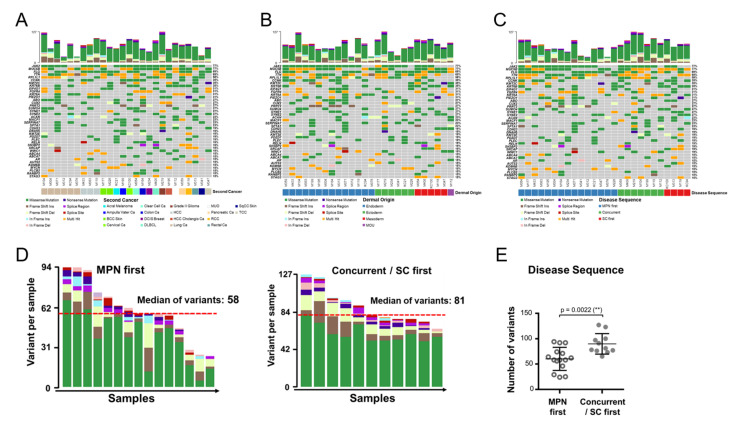
Comparisons on WES data of MPN patients with SCs. (**A**–**C**) Genes with variants in MPN with an SC stratified by cancer types (**A**), dermal origins (**B**), or disease sequence (**C**). Each stratification is represented by a corresponding color box shown at the bottom. (**D**) Subtypes and numbers of genomic variants stratified by the time sequence between the onset of MPN and SC. (**E**) Comparison of the numbers of variants based on disease sequence. **: *p* < 0.01, by Student’s *t*-test.

We next focused on the genetic changes in MPN patients with SCs. Probably hampered by the limited case numbers across different cancer types, we could not identify any unique pattern of genomic alteration among them (Figure 2A). When we subcategorized these SC tumors into different groups based on their dermal origins, we did not observe any noteworthy dissimilarity in their mutational profiles (Figure 2B) either. Looking further, we wondered whether the time sequence between the onset of MPN and an SC could be the result of discrepant genomic complexity. As demonstrated in Figure 2C, the genomic changes were less common in MPN-first cases when compared with those with concurrent or SC-first diseases. The median number of variants were 58 in MPN-first cases and 81 in the remaining samples (Figure 2D). The significantly lower average number of variants in MPN-first cases (Figure 2E) suggests that the genomic changes are more complex in MPN patients who had a preceding or concurrent SC.

Fertile genetic background is a popular theory for the acquisition of *JAK2*V617F mutation, as a particular *JAK2* gene haplotype, the GGCC or 46/1 haplotype, confers susceptibility to *JAK2*-mutated MPN [30,32,33]. To investigate the potential role of the 46/1 haplotype as a genetic predisposing factor in MPN–SC, we explored our MPN cohort to see whether its prevalence was discriminately biased between SC and control groups. Although there was a trend for the 46/1 haplotype to be more commonly observed in SC patients (76.9% versus 57.1% in the MPN control cases), the discrepancy was not statistically significant (*p* = 0.089). When probing into the number of genomic variants in MPN–SC patients stratified by the presence or absence of the 46/1 haplotype (Figure S1A), we could not observe any difference between them either.

The concept of a “poor prognosis” SC (PPSC) has been proposed in a collaborative European study exploring the survival outcome in MPN patients with an SC, which encompasses a variety of aggressive cancers that occur in the stomach, esophagus, liver, pancreas, lung, ovary, nervous system, and so on [34]. It makes us wonder whether a PPSC could be driven by more complex genomic variations. The case numbers of PPSCs and non-PPSCs were quite similar in our patient cohort. However, these patients could not be segregated by specific genetic background, as those with a PPSC harbored neither a specific mutation pattern nor a substantially higher number of genomic alterations (Figure S1B).

Overall, these findings indicate that genetic intricacy is probably inconsequential in imposing excessive risks in the development of SCs in MPN patients. However, when SCs do occur in MPN patients, it seems that the variation in genomic alterations is more significant in those with an SC preceding MPN as compared to MPN patients who develop SCs on a later date.

### 3.3. Genomic Alterations Are Allocated in Distinct Genes and Manifest Unique Co-Occurring Patterns in MPN with an SC

**Figure 3:**
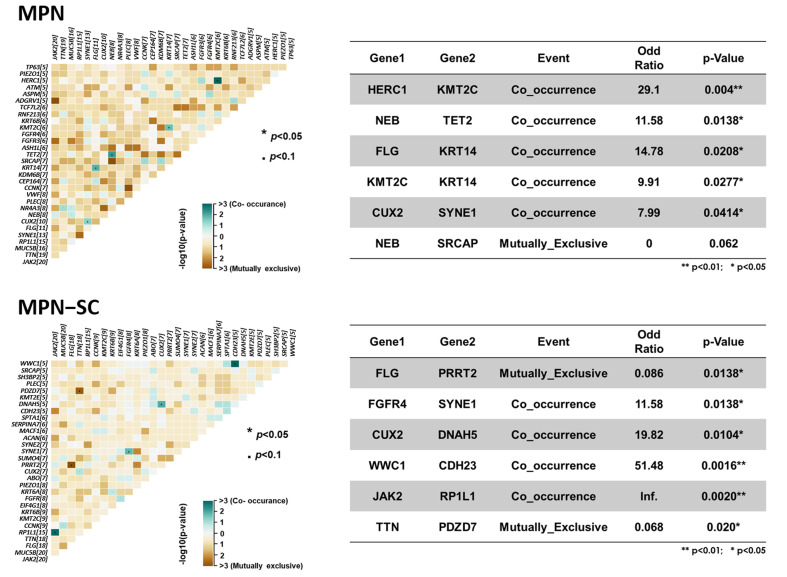
Patterns of co-occurrence and mutual exclusiveness of genes with variants between the two groups of MPN patients. Co-occurring and mutually exclusive gene pairs are highlighted in dark green and brown colors, respectively. Several interacting paired variants with statistical significance are outlined in the accompanying tables. Upper panel: MPN control group. Lower panel: MPN–SC group.

Looking into genes with alterations identified on WES, we noticed that some variants occurred concurrently, while others were mutually exclusive. As illustrated in Figure 3, the patterns of co-occurrence and mutual exclusiveness were discrepant between the two groups. Several interacting paired variants with statistical significance are outlined in the accompanying table (Figure 3). For example, alterations in *TET2* gene, one of the more commonly mutated genes in myeloid neoplasms, specifically co-occurred with *NEB* gene variations in control MPN patients, with the odds ratio for the co-occurrence being 11.6 times higher than that seen in MPN patients with SCs (*p* = 0.0138). However, *RP1L1* variants and *JAK2* mutation co-occurred prominently in MPN–SC patients, and compared to those without SCs, the odds ratio for such a co-occurrence was significantly high (*p* = 0.0020). The results suggest that MPN patients with SCs harbor genomic variances in distinct genes, among which a unique pattern of co-occurrence or mutual exclusiveness could be identified.

### 3.4. Critical Variant Replaces JAK2 as the more Prominent Disease Driver in MPN with an SC

**Figure 4:**
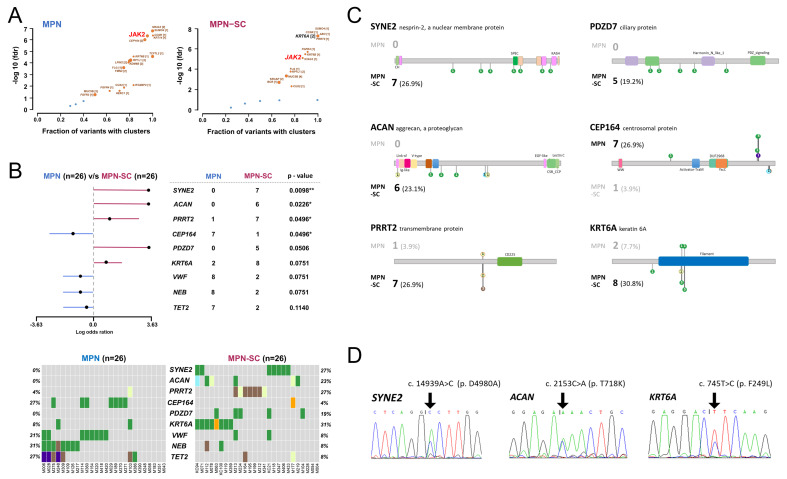
Significance and differences of various genes in MPN with an SC as compared with controls. (**A**) The significance and fraction of variants with clusters for each gene in both groups. Data were obtained with the OncodriveCLUST algorithm. (**B**) Genes differentially altered between the two groups of MPN patients. (**C**) Comparative lollipop diagrams of several key genes between the two groups of patients. Upper: MPN control group. Lower: MPN–SC group. Each lollipop indicates the position where a specific mutation occurs. Case numbers are shown in bold, and the prevalence of variants of a specific gene is described in parentheses. (**D**) Validation with Sanger sequencing. Three representative genes (*SYNE2*, *ACAN*, and *KRT6A*) exhibiting variants predominantly seen in MPN patients with SCs on WES were selected, and DNA samples from involved patients were subjected to validation through Sanger sequencing. Arrows indicate the positions of the mutations. *: *p* < 0.05; **: *p* < 0.01.

It is widely known that gain-of-function mutations often cluster in specific protein regions that confer the mutated cells a clonal advantage [29]. To appraise genes with potential bias toward mutational clustering within the protein sequence, we used the OncodriveCLUST algorithm [29] to assess coding-silent mutations and to evaluate the significance and fraction of clustering variances for each gene. We found that *JAK2* mutation as the disease driver gene is not as dominant in MPN–SC as compared with that seen in the control group (Figure 4A), suggesting that other genomic variations might play more significant roles in the disease course that potentially leads to the development of an SC.

Through a comparison of genes with variants in both groups, we could identify several key genes that were differentially altered between the two groups (Figure 4B). To better discern, we also juxtaposed the genomic positions of individual mutations for each gene in both groups in lollipop diagrams (Figure 4C). As demonstrated in both figures, variants of *SYNE2, ACAN*, and *PRRT2* genes were more constantly seen in MPN patients with SCs, whereas *CEP164* was more commonly altered in control cases. Additionally, there were trends that both *PDZD7* and *KRT6A* genes were more frequently mutated in MPN–SC patients. We next used Sanger sequencing to confirm the findings on WES. While genetic alterations in *SYNE2*, *ACAN*, and *KRT6A* genes could be validated (Figure 4D), we could not identify the coding sequence mutation in the *PRRT2* gene (data not shown). The major *PRRT2* variant obtained from WES was an InDel mutation around c.640–641. However, as the sequence flanking *PRRT2* c.640 contains poly-C repeats, we assume that the aberrantly annotated variants around this region on WES analysis might be caused by homopolymer-associated errors.

To determine whether certain mutants are relevant to SC development, we adopted the ClinVar and COSMIC databases to characterize the significance of the top four genes (*SYNE2*, *ACAN*, *PDZD7*, and *KRT6A*) with alterations that were more preferentially allocated in the MPN–SC group. Almost all variants of *SYNE2*, *ACAN*, and *PDZD7* genes identified in our patients had not been shown to be of clinical importance with regard to cancer biology (Table S4). On the contrary, a major mutation in the *KRT6A* gene (*KRT6A* c.745T>C) has been linked to human carcinoma (including ductal carcinoma of breast and adenocarcinoma of lung and intestine) and predicted to be pathogenic in COSMIC (Table 2 and Table 3). Interestingly, when we looked into the comparative lollipop diagrams, we found that genomic variants of the *KRT6A* gene were highly clustered in one isolated region (Figure 4C), indicating a potential significance of clinical relevance that might warrant further functional studies to confirm its inherent role in cancer development. This finding is in contrast to what we saw in *SYNE2*, *ACAN*, and *PDZD7* (Figure 4C), three genes with diversely distributed variants that had just been deemed inconsequential in SC development in both ClinVar and COSMIC analyses. Importantly, the *KRT6A* c.745T>C mutation we identified in PB granulocytes was not detected in buccal cells (obtained through mucosal swab) on Sanger sequencing (Figure S2), implicating this one as being a somatic mutation in hematopoietic cells (rather than a germline alteration). In addition, looking back to data obtained with the OncodriveCLUST algorithm, we found that *KRT6A* gene mutation was a highly prominent disease driver in MPN with an SC (Figure 4A), a phenomenon not observed in MPN control cases. This suggests that this gene might play more significant roles in the disease course that potentially leads to the development of an SC. These findings may open up an avenue for the rational investigation of the genetic background and molecular pathogenesis that leads to the development of SCs in MPN patients.

### 3.5. Genomic Variants in MPN with an SC Are Enriched in Inflammation Signaling

**Figure 5:**
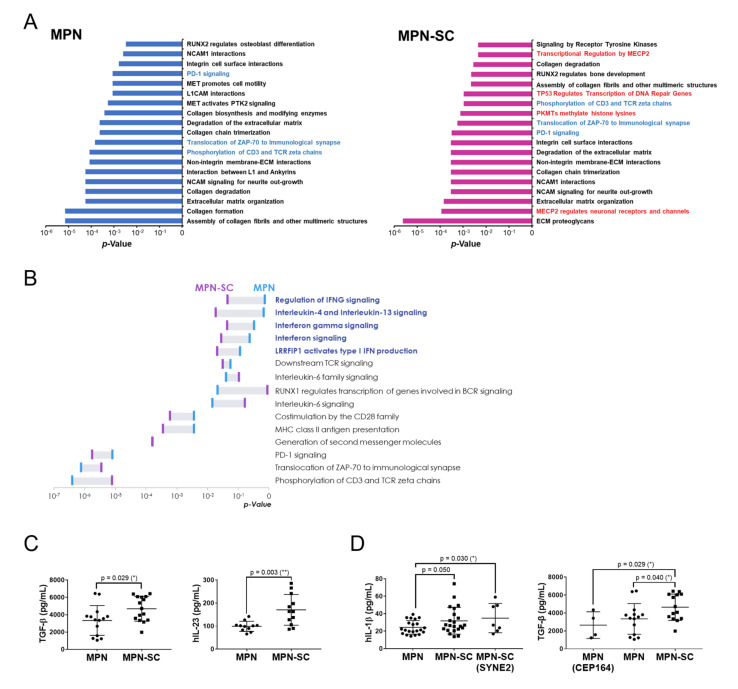
Gene ontology functional analysis and cytokine measurement in both groups of MPN patients. (**A**) Gene ontology functional analysis of genes with variants in the control group (left) and the SC group (right). Immune-related pathways are highlighted in light-blue color. Pathways pertinent to epigenetic regulation and genomic stability are highlighted in red. (**B**) Differentially involved pathways linked to the mutated genes in both groups. Some key inflammation pathways enriched in SC samples are highlighted in blue. The Reactome was applied for this analysis. (**C**) Comparison of TGF-β and IL-23 levels in the two groups of MPN patients. (**D**) Comparison of IL-1β and TGF-β levels in the indicated groups of MPN patients. *: *p* < 0.05; **: *p* < 0.01 by Student’s *t*-test.

To assess whether genes with variants are particularly involved in specific pathways, we applied the Reactome for gene ontology functional analysis. We found that genes involved in immune-related pathways were significantly altered in both groups (Figure 5A). Furthermore, we identified that in the SC group, the genes with variants were also peculiarly enriched in epigenetic regulation and genomic stability, such as DNA methylation, TP53-regulated transcription, and DNA repairs (Figure 5A). The results suggest that epigenetic deregulation and genomic instability might play a role in the development of SCs in MPN patients.

As discussed earlier, systemic inflammation could be the key predisposing factor for an MPN-associated SC [15]. Therefore, we specifically focused on inflammation. We found that the genomic variations in SC samples were rich in some key inflammation pathways, especially interferon- and interleukin-related signaling (Figure 5B). Looking further, we compared the plasma cytokine levels between the two groups of patients. Among a handful of cytokines, both TGF-β and IL-23 levels were significantly elevated in MPN patients with SCs (Figure 5C). To discern whether genetic alterations could have some impacts on cytokine levels, we also performed subgroup analysis stratified by differentially altered variants. As mentioned earlier, mutations in the *SYNE2* gene were more commonly seen in those with SCs. We observed that, compared to control cases, MPN–SC patients harboring *SYNE2* variants had significantly higher plasma IL-1β levels (Figure 5D). However, *CEP164* variants were primarily identified in control cases. We noticed that the level of TGF-β was further decreased in control cases harboring *CEP164* variants as compared to that in MPN–SC patients (Figure 5D). These data suggest that inflammation could be indispensable in the development of SCs in MPN patients. It is plausible that the inflammatory milieu might be partially attributable to the genomic variants in these patients. However, hampered by the limited case number in our study, these speculations definitely warrant confirmation from in vivo experiments and validation from other similar cohorts.

## 4. Discussion

Despite accelerated clarification of molecular pathogenesis leading to secondary AML evolution in MPN, the specific genetic events governing SC development in these patients remain elusive and unexplored. Studies in this aspect are mostly epidemiological, which mainly focus on clinical risk factors, and contradictory reports are not uncommon [3,4,5,6,7]. Five non-driver mutations (*ASXL1, TET2, SETBP1, EZH1*, and *TP53*) have been evaluated in a case-control study involving 142 MPN patients, but none are strongly linked with an SC in MPN [14]. Through use of WES, we were able to provide a sneak peek at the genetic heterogeneity of MPN patients with SCs. Importantly, this work unveils distinct genomic variant profiles involving epigenetic regulation and inflammation pathways that could be relevant in the development of SCs in MPN patients.

Driver mutations in MPN result in constitutive activation of the JAK-STAT pathway, which leads to the release of a plethora of cytokines and resultant systemic inflammation [35]. It is also well known that chronic inflammation can promote tumorigenesis by igniting abnormal cellular proliferation, nurturing angiogenesis, accelerating malignant transformation, and enhancing eventual dissemination of cancers [36]. Furthermore, researchers have implied that vascular endothelial growth factor and TGF-β, both highly expressed in MPN patients, can induce qualitative and quantitative defects in the immune system [37,38]. Therefore, MPN, as an inflammation disorder, imposes an excessive risk of second cancers in afflicted individuals. The sustained inflammatory state, coupled with a defective tumor immune surveillance, creates a permissive niche that potentially elicits and drives the development of an SC [15]. The increased SC risk is present not only after the establishment of MPN but also prior to the MPN diagnosis [39]. Our work attests to the theory by demonstrating that MPN–SC patients harbor more genomic variants enriched in key inflammatory pathways (Figure 5B), indicating that second cancers are liable to evolve in MPN patients with more pronounced inflammation. Substantially, the comparably higher serum cytokine levels (especially TGF-β) in MPN–SC patients (Figure 5C) further demonstrate the probability of inflammation-imposed tumorigenesis in MPN.

Among the four mutated genes that were more significantly enriched in MPN–SC patients, we found that *KRT6A* probably carried the most clinical significance (Figure 4 and Table 2 and Table 3). The fact was strongly supported by several aspects, including the clustering of mutants (hotspot mutations) and the reported link with human carcinoma when annotated in COSMIC. Remarkably, its role as a disease driver was deemed more prominent in MPN patients with SCs when analyzed with OncodriveCLUST. Unlike traditional analytic methods, OncodriveCLUST provides critical benefits in identifying candidate genes that are commonly missed by criteria based on frequency and functional impact. Through incorporation of coding-silent mutations for modeling construction, this method helps discern disease-specific driver mutations. In our study, we unequivocally identify *JAK2* mutation as the key disease driver in the traditional MPN patients without SCs, when using OncodriveCLUST analysis. Yet, in the MPN–SC group, *KRT6A* mutation played more prominent roles than *JAK2*V617F did in the propagation of diseases. This suggests that the genetic and/or environmental background for both MPN and a second cancer to evolve is different from that for MPN alone. *KRT6A* has been shown to mediate the activity of tumor-associated macrophages (TAMs) in cancers [40], which could possibly be involved in the link between inflammation and cancers. Eight of our MPN–SC patients harbored *KRT6A* mutations. Among them, c.745T>C constituted the most prevalent mutation and was found in six cases (Table 2): two patients with lung cancer, two with hepatoma, one with pancreatic cancer, and one with metastasis of unknown origin. As mentioned earlier, the mutation was confirmed to be somatic (Figure S2), implicating its sole presence in the hematopoietic cells. In the COSMIC database, *KRT6A* c.745T>C mutation is considered pathogenic and has been described in various cancers, including adenocarcinoma of lung. Furthermore, in both lung and pancreatic cancer, up-regulation of *KRT6A* has been associated with an aggressive tumor phenotype and an adverse clinical outcome [40,41]. Based on the known function of this gene, it is conceivable that the *KRT6A* variant, building on a collaborative feedback loop from the inflammatory niche endued by MPN, creates a more permissive microenvironment that could be substantially favorable for the establishment and expansion of the SC clone. However, the major caveat to keep in mind here is that *KRT6A* c.745T>C mutation in our MPN patients is found in PB granulocytes, whereas the same mutant reported in COSMIC or the literature mostly exists in the primary tumor samples. Whether granulocytes (or maybe the closely related myeloid lineage cells, i.e., TAMs) harboring this mutation contributes to tumorigenesis remains to be seen. Additionally, it is not immediately clear whether *KRT6A* c.745T>C mutation could lead to an increased expression of the protein and a resultant alteration of the tumor phenotype. The results of our study nevertheless inspire potential interest in further exploration of the consequences of *KRT6A* c.745T>C mutation in myeloid cells with potential implications for inflammation as well as cancer growth in an MPN background.

The *JAK2* 46/1 haplotype, in complete lineage disequilibrium, has been a well-known germline risk variant for MPN [30,32,33]. It has been demonstrated that the *JAK2* 46/1 haplotype may lead to increased production of inflammatory cytokines and impaired immunity [42], both of which are key contributing factors in the development of cancers. Although the composition of genetic variants in our MPN–SC patients with or without the *JAK2* 46/1 haplotype was indistinguishable, we nevertheless identified a trend showing a remarkably higher proportion (76.9%) of MPN–SC patients harboring this haplotype than those without SCs (57.1%). Not surprisingly, based on data obtained from the Taiwan Biobank, both numbers are higher than the reported frequency of 25.1% in the healthy Taiwanese population (see Supplementary Materials). Rumi et al. examined the risk of lymphoid neoplasm in patients with MPN and concluded that the *JAK2* 46/1 haplotype is not a genetic predisposing factor [3]. However, they barely enrolled 11 index cases (against 678 MPN controls). In fact, studies exploring this haplotype as a potential link between MPN and solid tumors have been lacking. It is indeed difficult to make sound conclusions based on our work incorporating only a few dozen MPN–SC cases. Considering that the *JAK2* 46/1 haplotype-conferred inflammatory milieu could well be advantageous for tumorigenesis, we anxiously await future validation from cohorts containing more MPN–SC patients.

Our study does not completely solve the enigma surrounding the genetic basis of MPN-associated SCs. Based on our work, epigenetic deregulation and genomic instability are more prominent in MPN–SC patients, whereas other factors, such as genetic complexity, types of driver mutation, and MPN subtypes, are all irrelevant to SC development. This is in contrast with previous reports showing that both *JAK2*V617F mutation and single-nucleotide polymorphism of the *TERT* gene impose excessive risk for the development of solid cancers in MPN patients [43]. We did find an interesting link between driver mutations and SC subtypes, yet the observation of a stronger association between *JAK2* mutation-endodermal tumors and between *CALR* mutation-mesodermal tumor could be biased because of the limited case number in our study. However, in our MPN patients who did develop second cancers, we noticed that those with preceding or concurrent SCs had significantly more genetic variants, suggesting that genomic complexity could factor in the growth rate of SC tumors. This could also imply that the severity of MPN has little role in the pace of SC tumorigenesis.

Despite our in-depth exploration of genomic alterations in MPN patients with SCs, the clinical applicability of our study is admittedly restrained in several aspects. Often, WES studies could unearth variants that are either erroneous or of little clinical significance. To avoid these flaws, we used Sanger sequencing to validate the presence of key mutants and exclude the inaccurate variants (such as *PRRT2*). We also meticulously applied various tools to remove low-quality reads, minimize artifacts, and discriminate synonymous variants. Furthermore, the limited case number of MPN–SC patients could make the analytical outcome ambiguous. We did have that in mind but just intended to provide an initial glimpse of MPN–SC genetics. Additionally, our inability to procure paired samples from SC tumors and their adjacent tumor tissues might have impeded our validation of key drivers in second cancers, as studies on the tumor microenvironment would informatively demonstrate our theory on the inflammation-imposed development of SCs. Lastly, tumorigenesis usually takes quite a long time, which makes the causal relationship between MPN-induced inflammation and an SC rather indistinct. Coupled with the genomic complexity we commonly see in cancers, it is therefore extremely difficult to appraise the function of a particular gene in the development of an SC in a simulated in vivo experimental model constructed with an MPN background.

## 5. Conclusions

In conclusion, our work provides a genomic map that silhouettes the genetic basis for SC tumorigenesis in MPN patients. Although the pattern of genomic variations in MPN–SC patients is not disparate from that of control cases, there is preferential involvement of variants in the inflammatory pathways in these patients, a finding supported by the increased plasma levels of two inflammatory cytokines TGF-β and IL-23. We also identified genetic variants that might potentially replace *JAK2*V617F as the more prominent disease driver in MPN with an SC. The obtained information could potentially advance the progress in our dissection of the molecular pathogenesis that leads to the development of SCs in MPN patients.

## Figures and Tables

**Table 1:** Baseline characteristics of MPN patients.

Variables	MPN without SC (*n* = 193)	MPN with SC (*n* = 27)	*p*-Value
** Age ^#^ (mean ** ** ± SD) **	60.8 ± 16.8	70.2 ± 14.6	0.006
** Male gender **	88 (45.6%)	16 (59.3%)	0.183
** Diagnosis ^†^**			0.774
** PV **	61	6	
** ET **	94	16	
** PrePMF **	7	1	
** PMF **	31	4	
** Driver mutation **			0.448
***JAK2*V617F **	143	19	
*** JAK2 * Exon 12 **	1	1	
***CALR***	23	4	
*** MPL ***	6	0	
** Triple negative **	20	3	
** Tumor origin **			NA^+^
** Ectoderm **	-	6	
** Mesoderm **	-	6	
** Endoderm **	-	14	
** MUO ^^^**	-	1	
** Secondary MF **	21 (13%) *	3 (13%) *	1.000
** Thromboembolism **	52 (26.9%)	9 (33.3%)	0.487
** Major bleeding history **	31 (16.1%)	8 (29.6%)	0.084
** White cell count, ×10^9^/L **	14.88 ± 10.95	14.90 ± 8.11	0.992
** Hemoglobin, g/dL **	14.2 ± 3.7	12.8 ± 3.3	0.062
** Platelet, ×10^9^/L **	619 ± 375	725 ± 361	0.172
** Splenomegaly **	103 (58.5%) *	12 (50%) *	0.343

^#^ Age at diagnosis of MPN; ^†^ PV: polycythemia vera; ET: essential thrombocythemia; PMF: primary myelofibrosis; ^^^ MUO: metastasis of unknown origin; ^+^ NA: not available; * estimation of the percentages was based on those with available data.

**Table 2:** Variants of the *KRT6A* gene identified in our MPN patients with second cancers.

Coding Sequence Mutation	Amino Acid Mutation	Case Number	Cancer Type in Enrolled MPN Patients ^#^
c.721_722delGGinsAA, c.745T>C	p.Gly241Asn (G241N), p.Phe249Leu (F249L)	3	MUO, HCC, Pancreas
c.745T>C	p.Phe249Leu (F249L)	2	Lung, Lung
c.745T>C, c.721_722insAGAGA, c.716_717insAAGACAGAAGACAGACACACACAGTGAGAGAGACAGA	p.Phe249Leu (F249L), p.Gly241fs *29, p.Gly241fs *25	1	HCC
c.721_722delGGinsAA	p.Gly241Asn (G241N)	1	Lung
c.418G>A	p.Val140Ile (V140I)	1	Cervix

^#^ MUO: Metastasis of unknown origin; HCC: Hepatocellular carcinoma.

**Table 3:** Annotation of *KRT6A* variants in COSMIC.

Coding Sequence Mutation	Amino Acid Mutation	COSMIC	FATHMM Prediction	Cancer Type in COSMIC
c.745T>C	p.Phe249Leu (F249L)	COSM1739982	Pathogenic (score 0.86)	14 Carcinoma, 1 Lymphoma
c.418G>A	p.Val140Ile (V140I)	COSM1239293	Neutral (score 0.09)	4 Carcinoma, 1 Lymphoma
c.721_722delGGinsAA	p.Gly241Asn (G241N)	Not found		
c.721_722insAGAGA	p.Gly241fs *29	Not found		
c.716_717insAAGACAGAAGACAGACACACACAGTGAGAGAGACAGA	p.Gly241fs *25	Not found		

## Data Availability

All relevant data are available from the corresponding authors upon reasonable request.

## Supplementary Materials

The following supporting information can be downloaded at https://www.mdpi.com/article/10.3390/cancers14143435/s1: Supplementary Methods, Figure S1. Genes with variants in MPN with SC stratified by 46/1 haplotype or by the prognosis of the SC subtypes; Figure S2. Assessing the nature of the *KRT6A* c.745T>C mutation in MPN patients; Table S1: Clinical profiles of enrolled MPN patients with second cancer; Table S2: Comparison between tumor origins and either MPN subtypes or driver mutations; Table S3: Baseline characteristics of MPN patients enrolled in this study; Table S4: Variants of *SYNE2*, *ACAN*, and *PDZD7* genes identified in our MPN patients with second cancers; Table S5: Information on PCR primers used in this study.
