# Supplementary material for: The Genomic Landscape in Philadelphia-Negative Myeloproliferative Neoplasm Patients with Second Cancers
Source: Cancers (Basel). 2022 Jul 14;14(14):3435. doi: 10.3390/cancers14143435 (PMC9316742; doi:10.3390/cancers14143435)
Supplement: Supplementary file 1 [file cancers-14-03435-s001.zip › cancers-1793361-supplementary.pdf]

Supplementary Materials

The Genomic Landscape in Philadelphia-Negative Myeloproliferative Neoplasm Patients with Second Cancers

Hsu *et al*.

Supplementary Figures and Tables

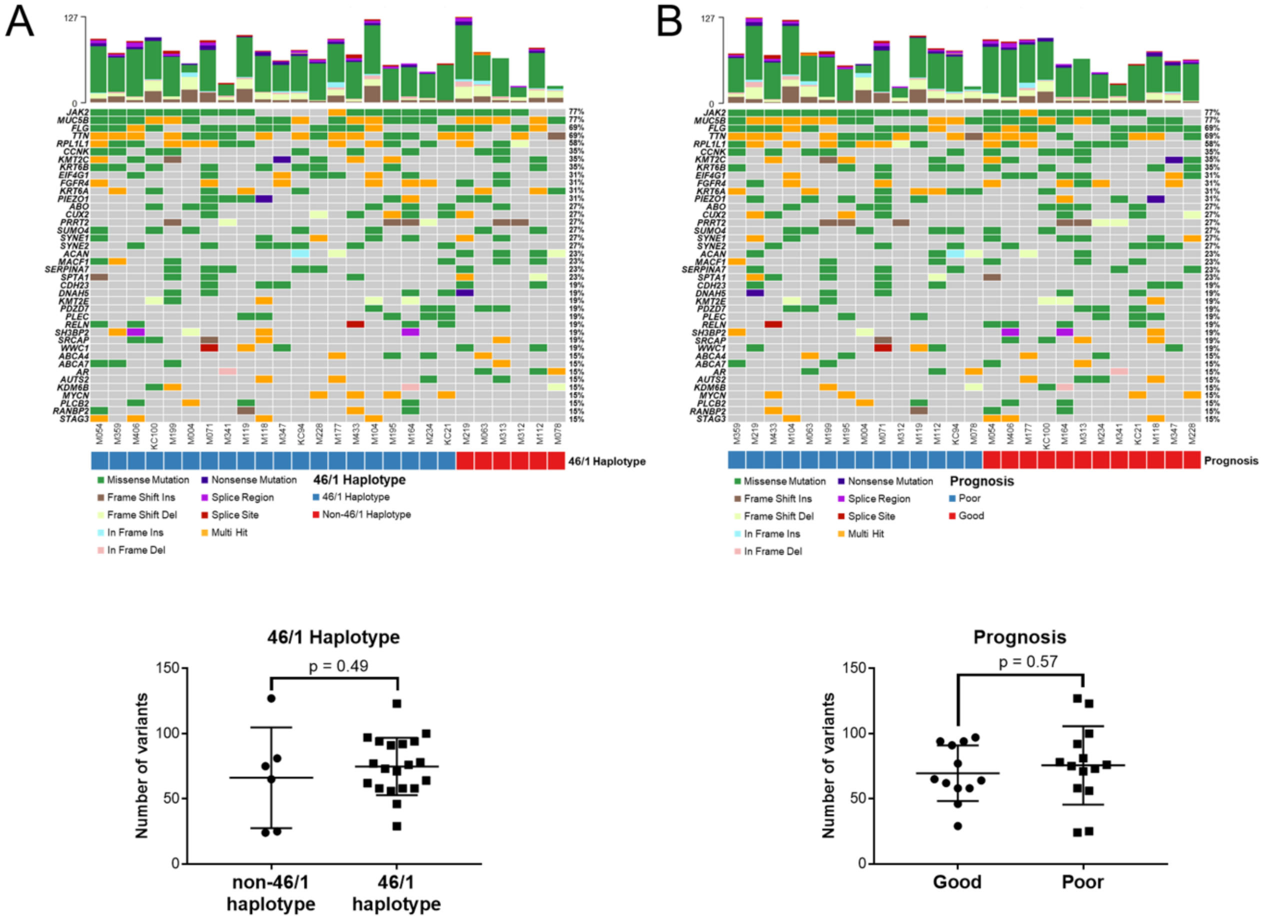

Supplementary Figure S1. Genes with variants in MPN with SC stratified by 46/1 haplotype or by the prognosis of the SC subtypes. (A-B) Comparative illustration of genes with variants in individual MPN-SC patients stratified either by the presence or absence of 46/1 haplotype (A) or by the prognosis of the SC subtypes (B). Each stratification was represented by a corresponding color shown at the bottom. Prognosis-driven subcategorization of SC types was based on the stratification proposed by Marchetti M *et. al.*, as the “poor prognosis” SC (PPSC) encompassed a variety of aggressive cancers that occur in the stomach, esophagus, liver, pancreas, lung, ovary, nervous system, and so on [1].

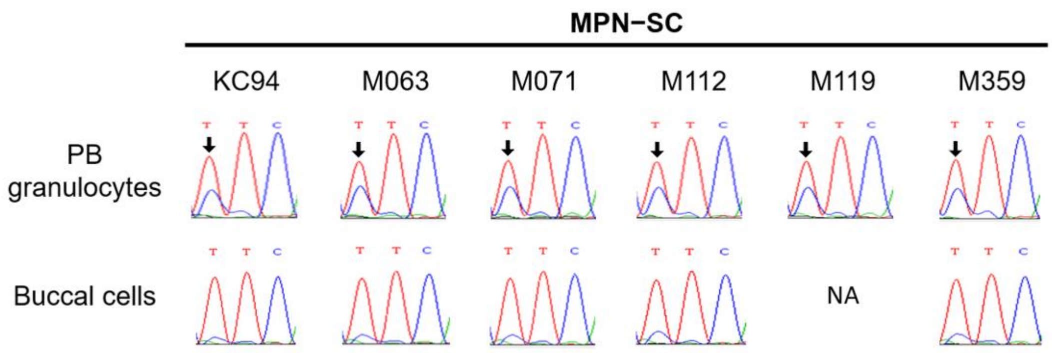

Supplementary Figure S2. Assessing the nature of the *KRT6A* c.745T>C mutation in MPN patients. DNA from PB granulocytes and buccal mucosal cells (germline DNA) was extracted and subjected to Sanger sequencing respectively. The mutation was detected in granulocytes but not in buccal cells. The result confirms this mutation being somatic in nature. Arrows indicate the positions of the mutation.

Supplementary Table 1. Clinical profiles of enrolled MPN patients with second cancer

ID	Age[1]	Sex	Dx	Driver[2]	SC Type[3]	Origin	Disease sequence[4]
1	84.1	F	PMF	V617F	SqCC (Skin)	Ectoderm	MPN first
2	70.1	M	ET	*CALR*	BCC (Skin)	Ectoderm	MPN first
3	45.5	M	ET	V617F	BCC (Skin)	Ectoderm	MPN first
4	43.1	F	PV	V617F	DCIS (Breast)	Ectoderm	MPN first
5	67.4	M	ET	V617F	Colon	Endoderm	MPN first
6	57.6	F	PV	V617F	Acral Melanoma	Ectoderm	MPN first
7	78.5	M	PrePMF	V617F	DLBCL	Mesoderm	Concurrent
8	78.0	M	PMF	TN	MUO		Concurrent
9	92.9	M	PMF	V617F	HCC	Endoderm	Concurrent
10	75.9	M	PMF	V617F	Grade II Glioma	Ectoderm	Concurrent
11	80.2	M	ET	V617F	HCC	Endoderm	MPN first
12	65.5	M	PV	V617F	Lung	Endoderm	Concurrent
13	71.8	M	PV	V617F	Rectum	Endoderm	SC first
14	54.0	M	PV	V617F	HCC + Cholangio Ca	Endoderm	MPN first
15	67.3	F	ET	TN	Lung	Endoderm	MPN first
16	62.9	F	ET	V617F	Lung	Endoderm	MPN first
17	74.0	F	ET	V617F	Lung	Endoderm	MPN first
18	80.3	F	ET	V617F	Clear cell carcinoma	Mesoderm	MPN first
19	87.6	F	ET	V617F	HCC	Endoderm	MPN first
20	74.6	M	ET	*CALR*	TCC	Mesoderm	MPN first
21	70.6	M	ET	*CALR*	RCC	Mesoderm	SC first
22	45.9	M	ET	V617F	Lung	Endoderm	MPN first
23	88.7	M	ET	V617F	Ampulla Vater	Endoderm	Concurrent
24	89.7	M	ET	V617F	Pancreas	Endoderm	Concurrent
25[5]	80.8	F	ET	*CALR*	RCC	Mesoderm	MPN first
26	68.9	F	ET	TN	Lung	Endoderm	SC first
27	40.5	F	PV	Exon 12	Cervix	Mesoderm	SC first

[1] Age: Age in years

[2] Driver: Driver mutation: V617F: *JAK2*V617F; *CALR*: *CALR* Exon 9; TN: Triple negative; Exon 12: *JAK2* Exon 12

[3] SC: Second cancer: SqCC: Squamous cell carcinoma; BCC: Basal cell carcinoma; DCIS: Ductal carcinoma in situ; DLBCL: Diffuse large B cell lymphoma; MUO: Metastasis of unknown origin; HCC: Hepatocellular carcinoma; Cholangio Ca: Cholangiocarcinoma; TCC: Transitional cell carcinoma; RCC: Renal cell carcinoma

[4] Disease sequence: We considered MPN and SC as concurrent diseases if the diagnoses were made within six months of each other.

[5] The DNA sample of this case failed quality control scrutinization and was excluded from WES analysis.

Supplementary Table 2. Comparison between tumor origins and either MPN subtypes or driver mutations

	Endoderm	Mesoderm	Ectoderm	*p*-value
**MPN subtype**				0.276
**PV**	3	1	2	
**ET**	10	4	2	
**PMF/PrePMF**	1	1	2	
**Driver mutation**				0.054*
**JAK2**	12	3	5	
**CALR**		3	1	
**TN**	2			

* *JAK2* vs. *CALR* mutation: $p = 0.027$

Supplementary Table 3. Baseline characteristics of MPN patients enrolled in the current study

**Variables**	**With second cancer (n=26)**	**MPN Control (n=26)**	***p*-value**
**Age#, years**	69.8 ± 14.8	71.7 ± 12.6	0.632
**Male (%)**	16 (61.5%)	16 (61.5%)	1.0
**Diagnosis**			1.0
**PV**	6	6	
**ET**	15	15	
**PrePMF**	4	4	
**PMF**	1	1	
**Driver mutation**			1.0
***JAK2*V617F**	19	19	
***JAK2* Exon 12**	1	1	
***CALR* Exon 9**	3	3	
MPL	0	0	
**Triple negative**	3	3	
***JAK2*V617F AB^**	57.9% ± 33.4%	56.1% ± 30.2%	0.867
**MF transform, No. (%)**	2 (7.7%)	1 (3.8%)	1.0
**Hydroxyurea exposure**			0.579
**Yes**	12	14	
**No**	14	12	
**Anagrelide exposure**			1.000
**Yes**	7	7	
**No**	19	19	
**Ruxolitinib exposure**			0.638
**Yes**	2	3	
**No**	24	23	
**HU exposure time***	58.4 ± 65.1	60.5 ± 57.3	0.930
**Follow-up duration***	84.6 ± 70.6	75.8 ± 64.9	0.644

Age at diagnosis of MPN; mean ± standard deviation

^ AB: Allele burden

* Time in months; mean ± standard deviation; HU: hydroxyurea

Supplementary Table 4. Variants in *SYNE2*, *ACAN*, and *PDZD7* genes identified in our MPN patients with second cancers

	**Coding sequence mutation**	**Amino acid mutation**	**Case number**	**Cancer type***
**SYNE2**	c.7243A>G	p.Met2415Val	1	HCC
	c.10802A>G	p.Gln3601Arg	1	RCC
	c.12001_12002delTGinsCA	p.Trp4001Gln	1	Melanoma
	c.14939A>C	p.Asp4980Ala	1	DLBCL
	c.15740C>T	p.Thr5247Ile	1	Lung cancer
	c.18509C>T	p.Thr6170Met	1	Lung cancer
	c.19582C>A	p.Gln6528Lys	1	TCC
**ACAN**	c.151_152insTAAAGATAAG	p.Cys51fs*43	1	Lung cancer
	c.1666A>T	p.Arg556Trp	1	MUO
	c.2153C>A	p.Thr718Lys	1	Rectal cancer
	c.2842G>C	p.Ala948Pro	1	Glioma
	c.4150_4151insCTGCCCCTGGAGTAGAGGACATCAGCGGGCTTCCTTCTG	p.Ala1384_Ala1385insAlaProGlyValGluAspIleSerGlyLeuProSerGlyGluValLeuGluThrThr	1	Lung cancer
	c.4229_4230insTGCCTAGGAGTGAATGCACTAAAGCAGGGTTTTTCAAA	p.Ser1411fs*1	1	BCC
**PDZD7**	c.715T>A	p.Ser239Thr	1	HCC
	c.880T>G	p.Tyr294Asp	1	Lung cancer
	c.1267G>A	p.Ala423Thr	1	Melanoma
	c.1529G>A	p.Gly510Asp	1	Rectal cancer
	c.2126A>C	p.His709Pro	1	DCIS (Breast)

[*] Cancer type: the subtype of SC in our enrolled MPN patients. HCC: Hepatocellular carcinoma; RCC: Renal cell carcinoma; DLBCL: Diffuse large B cell lymphoma; TCC: Transitional cell carcinoma; MUO: Metastasis of unknown origin; BCC: Basal cell carcinoma; DCIS: Ductal carcinoma in situ.

Supplementary Table 5. Information on PCR primers used in this study

Primer	Sequence (5'-3')
**h_SYNE2_M2415V_F**	GTCTATAATCCAAGTAAACAGAAGAAACT
**h_SYNE2_M2415V_R**	GACTTTTCACTCTGTACAAGCAAGT
**h_SYNE2_Q3601R_F**	CTATTCAGGCATTTCATTAATTGTTGCA
**h_SYNE2_Q3601R_R**	TGGATACAAGACTGTGGGCCT
**h_SYNE2_W4001Q_F**	GCATATTTGCTGTGAGTGATTAAAACT
**h_SYNE2_W4001Q_R**	CACTGCACCACTAGAAAGGCT
**h_SYNE2_E4695A_F**	GATAACTATGTGTACTTCTCTGACTTA
**h_SYNE2_E4695A_R**	CTGTGTACCTGGCTCTTAGCAT
**h_SYNE2_D4980A_F**	TTAGGACTGATCAACATAGGAGGA
**h_SYNE2_D4980A_R**	CATTCGCCCAAGTCTTACCC
**h_SYNE2_T6170M_F**	CGGGTGTCAGGGGATAAAAGA
**h_SYNE2_T6170M_R**	GCTGGCTACTTAGAGGAAGTCA
**h_ACAN_T692P_F**	AAGGACGGGTCACTGGTAAGA
**h_ACAN_T692P_R**	GTAGGAAGACAGGGGTATGCA
**h_ACAN_R556W_F**	GTGCATCTACCAGCCCCTG
**h_ACAN_R556W_R**	CTTTGTATCGCCCTGTCAGCT
**h_CEP164_Q525R_F**	CACACACTGTACTCCCCAGTT
**h_CEP164_Q525R_R**	TACACACAGAGCAGCCACCA
**h_PDZD7_G510D_F**	CTCCTGAACCCCATTTTCAGACT
**h_PDZD7_G510D_R**	CAGTATGCACCCTCATCTGC
**h_PDZD7_S239T_F**	GTGGTGGGTGGCAGTGGA
**h_PDZD7_S239T_R**	TGCACCAGTCAAGGTCCTTGA
**h_PDZD7_Y294D_F**	GAAGTTGAAAGTTTACTCCCATCCT
**h_PDZD7_Y294D_R**	CGAGGTCTACCTATATGGCCAA
**h_PDZD7_A423T_F**	CTAAGCCCAAGGGTGGTCT
**h_PDZD7_A423T_R**	GATGTGAGACAGGTTTTGGACCA
**h_KRT6A_239_249_F**	GGTGCGGTTCCTGGAGCA
**h_KRT6A_239_249_R**	GTGCTCTTCATTTCCACGGACAT

Reference

1. Marchetti, M.; Ghirardi, A.; Masciulli, A.; Carobbio, A.; Palandri, F.; Vianelli, N.; Rossi, E.; Betti, S.; Di Veroli, A.; Iurlo, A.; et al. Second cancers in MPN: Survival analysis from an international study. *Am J Hematol* **2020**, *95*, 295-301, doi:10.1002/ajh.25700.
